# Monitoring the quality of Beluga fish (*Huso huso*) during cold storage using pH indicator patches: a comprehensive evaluation of chemical, microbial, and sensory changes

**DOI:** 10.1016/j.fochx.2026.103490

**Published:** 2026-01-04

**Authors:** Seyed Mohammad Ali Ebnetorab, Hamed Ahari, Seid Mahdi Jafari, Maryam Mizani, Seyed Amir Ali Anvar

**Affiliations:** a Department of Food Science and Technology, SR.C., Islamic Azad University, Tehran, Iran; b Department of Food Materials and Process Design Engineering, Gorgan University of Agricultural Sciences and Natural Resources, Gorgan, Iran; c Department of Veterinary Hygiene, SR.C., Islamic Azad University, Tehran, Iran

**Keywords:** Potassium Iodide (PubChem CID: 4875), Magnesium Oxide (PubChem CID: 14792), Boric Acid (PubChem CID: 7628), Sulfuric Acid (PubChem CID: 1118), Chloroform (PubChem CID: 6212), Bromocresol Green (PubChem CID: 6451), polycaprolactone (PubChem CID: 54078012), Gellan gum (PubChem CID: 60160837), Carboxymethyl chitosan (PubChem CID: 71306969), Intelligent packaging, Red cabbage, pH-sensitive indicator patches, Anthocyanin, Fish freshness monitoring, Chemical spoilage, Microbial spoilage

## Abstract

This study evaluated the effectiveness of pH indicator patches in monitoring the quality of Beluga fish (*Huso huso*) stored at 4 °C for 3, 7, and 14 days, assessing changes in chemical, microbial, and sensory properties. Three polymer solutions (G0, G1, G2) were used to create nanofibrous patches via electrospinning. The fish were stored, and chemical analyses (peroxide value (PV) and total volatile basic nitrogen (TVB-N)), sensory evaluation (texture, color, odor), and microbial counts (psychrophilic and thermophilic bacteria) were conducted. On day 3, G0 and G1 exhibited clearer color changes compared to G2, with G1 showing sharper shifts. By day 14, G0 and G1 exhibited a slight decrease in the a* value, whereas G2 showed greater variation. PV and TVB-N increased significantly, indicating oxidative spoilage and protein degradation. Sensory evaluation revealed a decline in color, odor, texture, and acceptability. The pH-sensitive patches effectively monitored fish quality, but further optimization is needed.

## Highlights

• pH-sensitive patches showed clear color transitions under varying pH conditions (5–9).
• G0 and G1 patches showed sharper color shifts, indicating greater sensitivity than G2.
• Significant color changes correlated with increases in TVB-N, reflecting protein degradation.
• Psychrophilic and thermophilic bacteria increased, supporting spoilage progression.
• Significant decline in color, smell, texture, and acceptability on day 14, confirming spoilage.

## Graphical abstract

**Figure:**
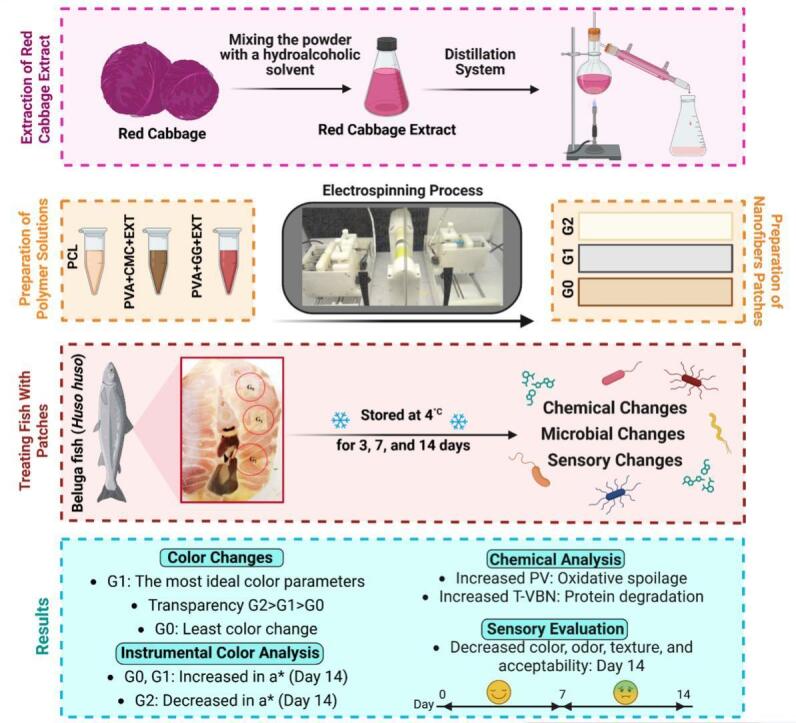

## 1 Introduction

The beluga sturgeon (*Huso huso*) is not only prized for its precious caviar but also regarded as a rich source of protein, omega-3 fatty acids, vitamins, and minerals (Dudu & Georgescu, 2024; Taheri et al., 2025). Compared to many other protein sources, such as red meat, beluga sturgeon meat is lower in both calories and fat, while having superior digestibility compared to various plant-based proteins (Taheri et al., 2025). Studies have demonstrated that regular consumption of beluga sturgeon can contribute to the prevention of chronic conditions such as diabetes, cardiovascular diseases, and certain types of cancer, making it a beneficial component of a nutritious and balanced diet (Barimani et al., 2021).

In recent years, several advanced approaches, such as electrochemistry, Raman spectroscopy, and hyperspectral imaging systems, have been developed to detect meat spoilage quickly (Ashiq et al., 2024; Cheng & Sun, 2015; Li, 2024). While these methods provide reliable results, they often require sophisticated equipment and highly skilled operators, making them impractical for real-time spoilage detection along the food supply chain (Pal & Kant, 2020). Therefore, the development of simple, cost-effective, and accessible strategies for on-the-spot spoilage monitoring is still a significant challenge (Kuswandi et al., 2022).

Microorganisms, including bacteria, fungi, and viruses, play a crucial role in human health (Dabbagh Moghaddam et al., 2024). Due to the broad pH range that affects microbial growth, enzymatic activity, and other spoilage factors, natural pH-sensitive color-changing indicators or patches can be utilized to monitor the quality of packaged food (Luo et al., 2023). These indicators exhibit measurable color changes as a result of shifts in pH caused by chemical reactions, microbial growth, or enzymatic activity in the food. Such indicators can be incorporated directly into the primary packaging, allowing them to be exposed to the product's environment and in contact with the food itself, providing a real-time, non-expert-friendly method for evaluating spoilage (Luo et al., 2023).

Generally, pH-sensitive visual indicators consist of two essential components: a solid matrix support and a colorant that responds to changes in pH (Luo et al., 2023). Thus, selecting and synthesizing non-toxic, pH-sensitive dyes and suitable solid matrix materials is critical for creating effective freshness indicators (Liu et al., 2022). A majority of existing patches are designed as hydrogels, which have a lower surface-to-volume ratio compared to nanofibers (Liu et al., 2022). Key limitations of hydrogel-based pH indicators for food packaging include their narrow pH range, potential for misinterpreting color changes, sensitivity to environmental factors (such as temperature, humidity, and pH), and instability of color shifts (Niemczyk-Soczynska & Sajkiewicz, 2025). Other concerns include incompatibility with packaging materials and limited sensitivity in detecting changes in the food matrix, especially in weakly acidic or alkaline conditions (Khan et al., 2024). The structural integrity of these patches during transport and storage is also a major challenge (Khan et al., 2024).

This study aimed to investigate the effect of pH indicator patches on the quality of Beluga fish (*Huso huso*) during storage at 4 °C. The study investigated the impact of chemical and microbial changes on different patches and how these effects are reflected in the color and pH variations of the fish meat over 3, 7, and 14 days. In addition to measuring chemical changes, such as peroxide value (PV) and total volatile basic nitrogen (TVB-N), sensory evaluations were conducted to assess the texture, color, and odor of the fish. Microbial counts of psychrophilic and thermophilic bacteria were also determined using the standard plate count method on plate count agar (PCA) medium. This study aimed to provide an innovative and effective approach for monitoring and controlling the quality of fish meat using pH indicator patches at 4 °C.

## 2 Materials and methods

### 2.1 Materials

All chemicals and reagents used in this study, including Methyl Red Indicator, Potassium Iodide, Magnesium Oxide, PCA, Boric Acid, Sulfuric Acid, and Chloroform, were procured from Sigma-Aldrich (Germany). Additionally, 96 % Ethanol and Sterile Distilled Water were sourced from Razi and Nikochemi Companies, Tehran, Iran, respectively. Bromocresol Green Indicator and Starch Powder were purchased from Merck, Germany. All materials were of high quality and used according to the experimental protocols' requirements.

### 2.2 Flowchart of the pH-sensitive patch preparation and testing

**Fig. 1:**
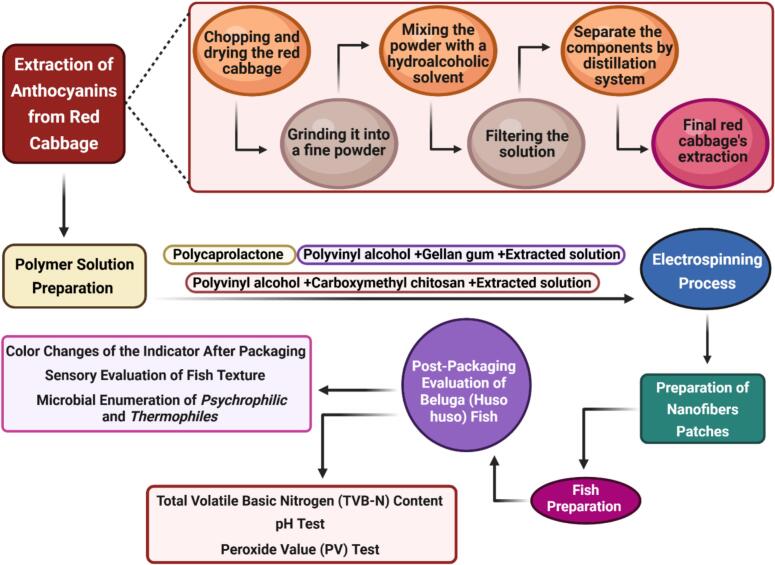
Flowchart illustrating the preparation and testing process of pH-sensitive indicator patches. This flowchart summarizes the key steps involved in preparing, electrospinning, and testing pH-sensitive indicator patches for monitoring the freshness of Beluga Fish fillets. The process includes the extraction of anthocyanins from red cabbage, preparation of polymer solutions (Group 1: Polycaprolactone in chloroform; Group 2: Polyvinyl alcohol and carboxymethyl chitosan with anthocyanin extract; Group 3: Polyvinyl alcohol and gellan gum with anthocyanin extract), electrospinning with different configurations (Group G0: Co-spinning of PCL and PVA/CMC/EXT solutions; Group G1: Co-spinning of PCL and PVA/GG/EXT solutions; Group G2: Single-nozzle electrospinning of PCL), preparing a patch for connection to the fish (2 × 2 cm patches applied to fish tissue), and post-packaging evaluation (color changes of patches analyzed visually and instrumentally, with ΔE calculated to assess spoilage). Abbreviations: polycaprolactone (PCL), carboxymethyl chitosan (CMC), red cabbage extraction (EXT), gellan gum (GG). (For interpretation of the references to color in this figure legend, the reader is referred to the web version of this article.)

Fig. 1 illustrates the entire process of preparing and testing the pH-sensitive indicator patches for monitoring the freshness of Beluga Fish fillets. The flowchart includes the key steps from the extraction of anthocyanins from red cabbage to the preparation of polymer solutions, the electrospinning process, and the final application of patches to the fish samples.

### 2.3 Extraction of anthocyanins from red cabbage

The red cabbage extract was prepared using the maceration method (Pourjavaher et al., 2017). Fresh red cabbage, purchased from a certified vegetable market in Tehran, Iran, was thoroughly washed, disinfected, and air-dried. 15 g of the powder was soaked in 50 mL of a 50:50 ethanol-water mixture. The mixture was stirred for 48 h on a magnetic hotplate and then kept in the dark at 4 °C for an additional 24 h. The extract was filtered, centrifuged at 2000 rpm for 10 min, and the supernatant was passed through Whatman #1 filter paper. Alcohol was removed using a distillation system (Shimi Azma Gostar, Iran) at 45–55 °C. The concentrated extract was stored in dark glass vials at 4 °C. To determine the extract's concentration, 5 mL was dried in an oven, and the dry matter content was calculated using formula 1 below:

(1) $$\mathrm{Dry}\;\text{Matter Concentration}\;\left(g/\mathrm{mL}\right)=\left(\text{Weight of petri dish after drying}–\text{Weight of empty petri dish}\right)/\text{Total extract volume}.$$

### 2.4 Polymer solution preparation and study design

Three distinct polymer solutions were prepared:

Group 1: This group consisted solely of polycaprolactone (PCL). To prepare the solution, 1 g of PCL granules (molecular weight 80,000) was dissolved in 10 mL of chloroform, resulting in a 10 % (w/v) polymer solution.

Group 2: This solution was composed of polyvinyl alcohol (PVA) and carboxymethyl chitosan (CMC). To prepare the solution, 0.45 g of PVA and 0.05 g of CMC were combined with 1 mL of the extracted solution (EXT) and 4 mL of distilled water. The resulting mixture, after thorough stirring, produced a polymer solution with a final concentration of 0.1 g/mL.

Group 3: This polymer solution combined PVA with gellan gum (GG). The preparation involved mixing 0.45 g of PVA and 0.05 g of GG with 1 mL of EXT and 4 mL of distilled water. After mixing, the resulting solution had a concentration of 0.1 g/mL.

### 2.5 Electrospinning process overview

Based on the available equipment's operational range and the information above, the parameters for electrospinning were optimized through repeated adjustments and testing. The tested ranges for the parameters were as follows: applied voltage (1–30 kV), needle-to-collector distance (5–20 cm), and solution feed rate (0.5–1 mL/h). The optimal parameters were selected based on fiber uniformity, alignment, and the absence of fiber breakage, with particular attention given to the jet breakup behavior and the formation of the Taylor cone at the needle tip. The more symmetrical the cone, the better the electrospinning results.

In general, increasing the voltage above 6 kV resulted in a stronger electric field and increased electrical charge on the solution, causing a larger volume of solution to be ejected from the needle tip. This led to an increase in the Taylor cone angle. Additionally, higher voltage created more rapid and intense stretching of the jet from the needle to the collector, resulting in a smaller Taylor cone and reducing or eliminating bead formation, ultimately improving the morphological characteristics of the nanofiber structure.

The effect of the polymer solution feed rate (flow rate) was found to be the least significant compared to the other process parameters. In this case, higher flow rates led to thicker fibers with larger diameters. As the flow rate increased, the number of beads in the nanofiber structure also increased, and in many cases, this caused an increase in fiber diameter due to the excessive solution being ejected from the needle tip, inadequate time for drying and solvent evaporation,and lower applied stretching forces. On the other hand, at lower flow rates, the polymer solution had sufficient time for solvent evaporation and polarization, resulting in more stretched jets and the production of finer fibers. Therefore, a lower flow rate was found to be ideal for producing finer fibers with no bead formation.

Increasing the needle-to-collector distance led to a longer jet travel time, which significantly increased the fiber diameter due to the reduction in electric field strength and the formation of beads. Additionally, at very short distances, the solvent did not have enough time to fully evaporate before reaching the collector, resulting in the formation of wet fibers. Therefore, the needle-to-collector distance was optimized to ensure adequate time for fiber drying before collection.

After several rounds of trial and error, the optimal electrospinning parameters were determined as follows: 15 kV for applied voltage, 0.5 mL/h for solution flow rate, and 10 cm for needle-to-collector distance. These settings resulted in the best quality spun fibers, with optimal morphology and minimal bead formation.

The electrospinning was performed in three different experimental setups, as described below:

Group G0: In this group, Solution S1 (PCL) was loaded into pump 2, while Solution S2 (PVA/CMC/EXT) was loaded into pump 1.

Group G1: Solution S1 (PCL) was placed in pump 2, and Solution S3 (PVA/GG/EXT) was loaded into pump 1 for this setup.

Group G2: Only Solution S1 (PCL) was used in this group, which was placed in pump 2.

The electrospinning procedure for Groups G0 and G1 involved co-spinning (using two pumps), while Group G2 employed a single-nozzle electrospinning method. The experiments were conducted under ambient temperature with a relative humidity of 49 ± 2 %. Based on these conditions, the most suitable parameters were chosen to achieve the desired nanofiber properties (Terra et al., 2021).

### 2.6 Fish preparation and experimental conditions

A total of one fresh Beluga (*Huso huso*) fish weighing 20 ± 1 kg was purchased from the sturgeon farming pond of Pearl Qorog (Gilan, Iran). After washing, the fish were divided into several sections from the middle part of the body. To precisely examine the behavior of the patches in each of the three groups (G0, G1, and G2), a 2 × 2 cm piece from each patch was directly placed on the tissue of each fish sample. The fish samples, along with the patches, were then wrapped in standard plastic film and stored at 4 °C for a period of 3, 7, and 14 days to assess the behavior and performance of the patches under cold storage conditions.

### 2.7 Post-packaging evaluation of Beluga (*Huso huso*) fish

#### 2.7.1 Color changes of the indicator after packaging

**Fig. 2:**
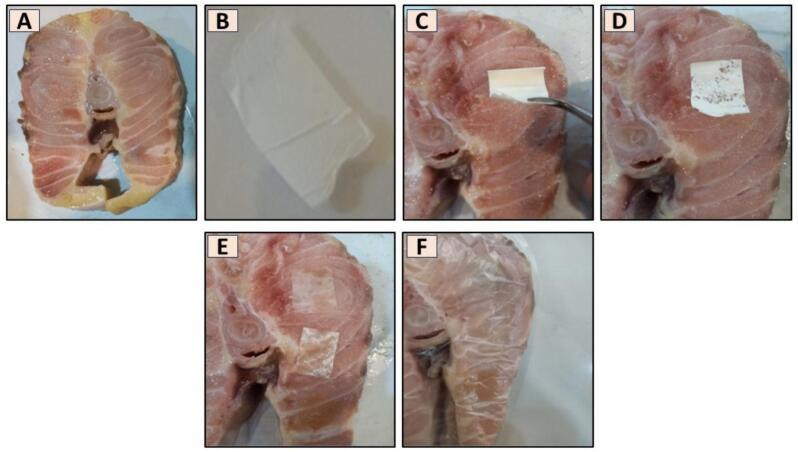
Cross-sectional cut of the mid-body region of a beluga sturgeon (*Huso huso*) (A). Preparation of the 2 × 2 cm patches, cut into small sizes for application (B). Direct attachment of the patches to the fish muscle tissue for analysis (C–E). Packaging of the fish meat covered with the various patches for further observation and testing (F).

The color changes of the patches attached to the surface of fish samples for all three groups (G0, G1, and G2) after packaging were evaluated visually and instrumentally on days 3, 7, and 14 (Fig. 2). For the instrumental evaluation, the patches were first dried and then analyzed. The color parameters (L* for lightness, a* for redness or greenness, and b* for yellowness or blueness) were measured using a Lovibond colorimeter, and the total color difference (ΔE) was calculated according to Eq. (1) (Tirtashi et al., 2019). Color changes at each pH were quantified using the total color difference (ΔE), calculated using the following formula (Moradi et al., 2019):

(1) $$\mathrm{\Delta E}=\sqrt{{\left(L^{*}-L_{0}^{*}\right)}^{2}+{\left(a^{*}-a_{0}^{*}\right)}^{2}+{\left(b^{*}-b_{0}^{*}\right)}^{2}}$$

#### 2.7.2 Sensory evaluation of Beluga (*Huso huso*) fish samples using a hedonic scale test

For sensory evaluation, different treatments of fish samples were assessed using a hedonic scale test with a five-point scale (5 represented the highest and 0 the lowest score). A score of 4 was considered the acceptance threshold for human consumption. The evaluation was conducted in four categories (texture, color, odor, and overall acceptability) on days 0, 3, 7, and 14.

#### 2.7.3 Microbial enumeration of *Psychrophiles* and *Thermophiles* in Beluga (*Huso huso*) fish

To determine the total counts of psychrophilic and thermophilic bacteria in Beluga fish fillet samples, the standard plate count method with surface plating on PCA agar was used on days 0, 3, 7, and 14. Briefly, PCA medium was prepared according to standard protocols, and 10 g of fish fillet was mixed with 30 mL of sterile distilled water (0.1 % concentration) in a stomacher bag. The mixture was homogenized for 2 min at 200 rpm, and 0.1 mL of the resulting suspension was surface-plated on PCA agar. The inoculated plates were incubated for 7 days at 10 °C to count psychrophilic bacteria, and for 24 h at 37 °C to count thermophilic bacteria. After the incubation period, the colony counts in 100 μL of the microbial suspension were calculated as Log CFU/mL.

### 2.8 Chemical test

#### 2.8.1 Total volatile basic nitrogen (TVB-N) content in Beluga (*Huso huso*) fish

The TVB-N content, which includes ammonia, dimethylamine, and trimethylamine, was determined by steam distillation during the storage of Beluga fish samples. 10 g of homogenized fish were mixed with 300 mL of distilled water, followed by the addition of 2 g of MgO and boiling stones. The mixture was heated until boiling and then distilled into a flask containing 2 mL of distilled water, 2 mL 2 % boric acid, and a few drops of methyl red and 0.1 % green bromocresol in ethanol. The distillation was conducted for 25–30 min. The resulting solution was titrated with 0.1 N sulfuric acid until a color change from yellow to dark pink, and the TVB-N content was calculated using Eq. (2) on days 0, 3, 7, and 14 (Mohammadalinejhad et al., 2020; Moradi et al., 2019)

(2) $$\mathrm{TVB}-N\;\text{Content}=\text{V1}\;\left(\text{volume of sulfuric acid used in titration}\;\left(\mathrm{mL}\right)\right)\times 14$$

#### 2.8.2 pH test in Beluga (*Huso huso*) fish

The pH values of the Beluga fish samples were measured after homogenizing 10 g of the sample in 90 mL of distilled water (on days 0, 3, 7, and 14). The pH was determined using a digital pH meter (Moradi et al., 2019).

#### 2.8.3 Peroxide value (PV) test in Beluga (*Huso huso*) fish

The PV was measured (on days 0, 3, 7, and 14) according to the Iranian National Standard No. 4179. 1 g of oil extracted from the fish sample (the fish was steamed and then the oil was separated under pressure) was weighed in a 250 mL Erlenmeyer flask. Approximately 25 mL of a chloroform-acetic acid solution (2:3 ratio of chloroform to acetic acid) was added to the contents of the flask, followed by 0.5 mL of saturated potassium iodide solution, 30 mL of distilled water, and a few drops of 1 % starch solution. It is worth noting that the same procedure was followed for the control sample, without adding fish oil. The amount of free iodine released was titrated with a 0.01 N sodium thiosulfate solution until the solution became colorless. The peroxide value was then calculated based on meq gr O₂/ Kg Fat using the following Eq. (3):

(3) $$\mathrm{Peroxide Value}\;\left(\mathrm{PV}\right)=\frac{\text{Volume of sodium thiosulfate used}\times \text{Normality of sodium thiosulfate}\;}{\text{Weight of the oil sample}}\times 1000$$

### 2.9 Statistical analysis

The experiment results are presented as mean ± standard deviation, based on triplicate data. The data were analyzed using a one-way analysis of variance (One-way ANOVA). Statistically significant differences among the mean values were determined using Duncan's multiple range post hoc test, applicable only when the overall treatment effect was significant. The relationships between patch color changes and spoilage indices were evaluated using Pearson's correlation coefficient. Statistical analyses were conducted using SPSS version 26 and GraphPad Prism version 6, with a significance level set at *p* ≤ 0.05 for data comparison.

## 3 Results

### 3.1 Color variation in patches post-packaging

#### 3.1.1 Visual observation of pH indicator patch color changes in G0, G1, and G2 groups post-packaging

**Fig. 3:**
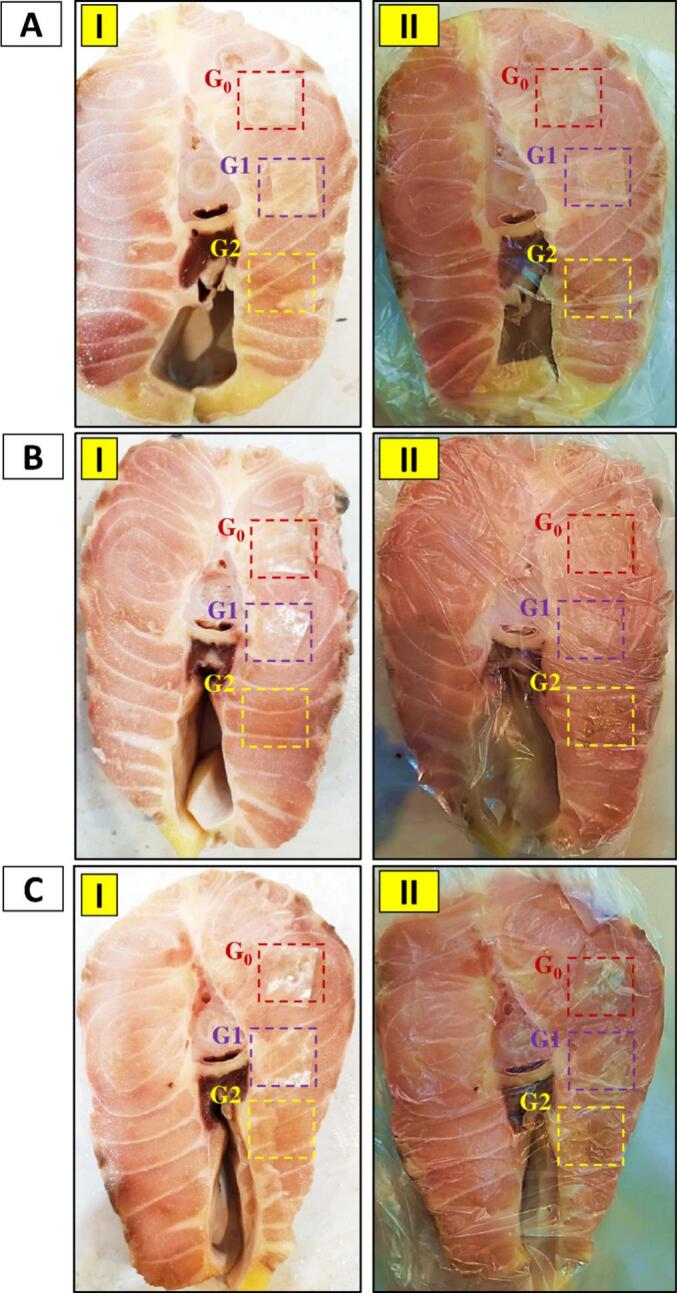
Treating the fish meat with the studied patches (G0, G1, G2) on days 3, 7, and 14. Visual evaluation of color changes in the patches after attachment to the fish surface on days 3 (AI), 7 (BI), and 14 (CI). Packaging the fish meat with the (G0, G1, and G2 patches on days 3 (AII), 7 (BII), and 14 (CII).Group G0 (PCL + (PVA/CMC/EXT)); Group G1 (PCL + (PVA/GG/EXT)); Group G2 (PCL). Abbreviations: polycaprolactone (PCL), carboxymethyl chitosan (CMC), red cabbage extraction (EXT). (For interpretation of the references to color in this figure legend, the reader is referred to the web version of this article.)

The visual observation of color changes in the designed patches across the three experimental groups (G0, G1, and G2) under various pH conditions (pH 5–9) was conducted using the naked eye after the patches were applied to the fish surface and the fish were subsequently packaged. The results are illustrated in Fig. 3.

As shown in Fig. 3, by the end of the third day, the color changes in the patches of all three groups were relatively clear, and the brightness of the patches remained high. This enhanced brightness allowed the color changes to be easily visible to the naked eye. The increased illumination made the color changes easily visible to the naked eye, with patches in the G0 and G1 groups showing greater visual clarity compared to the G2 patches. This difference is attributed to variations in the formulations and their ability to absorb amine compounds, which indicate an increase in pH levels.

This trend continued until the end of day 14, during which patches in groups G0 and G1 consistently showed higher visual clarity on the fish surface compared to the G2 patches. Among these, the G1 patches appeared sharper than those from group G0. After detachment of the patches from the fish surface, it was observed that all three patch types retained an acceptable level of visibility. However, the reduced clarity of G2 patches on the fish surface (on days 7 and 14) was likely due to their formulation, which consisted solely of PCL. PCL exhibited lower moisture sensitivity compared to the other formulations, thereby reducing the absorption of volatile amines. Furthermore, the anthocyanins in G2 patches were incorporated via immersion without additional protective measures, which may have contributed to the reduced visibility of these patches when applied to the fish surface.

#### 3.1.2 Instrumental color analysis of pH indicator patch color changes in G0, G1, and G2 groups post-packaging

**Fig. 4:**
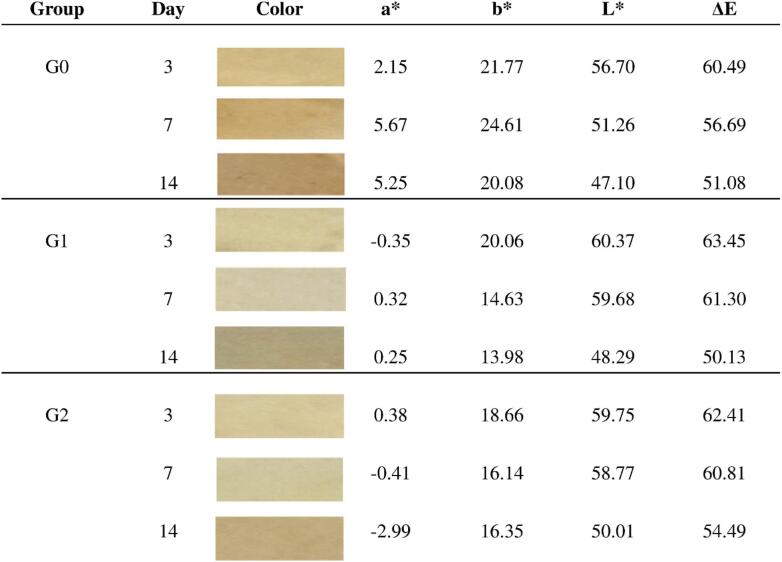
An overview of the reported colorimetric parameters in all three groups (G0, G1, and G2) at 4 °C on days 3, 7, and 14 is presented. As observed, all reported parameters in all three groups showed a decreasing trend, except for the parameter a*, which exhibited an increasing trend in the G0 and G1 groups, while it continued to decrease in the G2 group. This pattern indicates the lower capability of the PCL polymer matrix in protecting anthocyanins, likely due to its hydrophobic nature and limited barrier properties.

As observed on day 14, with the increase in pH and other contributing factors affecting color change, a slight decrease in the a* values was detected in both G0 and G1 groups. This reduction can be attributed to the absorption of amine compounds released from the fish surface, as well as the overall increase in pH. Moreover, the relatively higher a* value observed in group G0 indicates a lower sensitivity of this patch formulation to pH-induced color changes compared to group G1. Furthermore, the decrease in L* values in all three groups compared to day 7 suggests a reduced presence of chalcone precursors (carbinol pseudobases) and their subsequent transformation into darker brown pigments, indicating the degradation of yellow chalcones. Accordingly, the final observed colors on day 14 were categorized as group G0 exhibited a dark yellowish-brown hue, group G1 showed a dull yellow-brown color, and group G2 displayed a light yellow-brown appearance (Fig. 4).

### 3.2 Chemical analyses

**Fig. 5:**
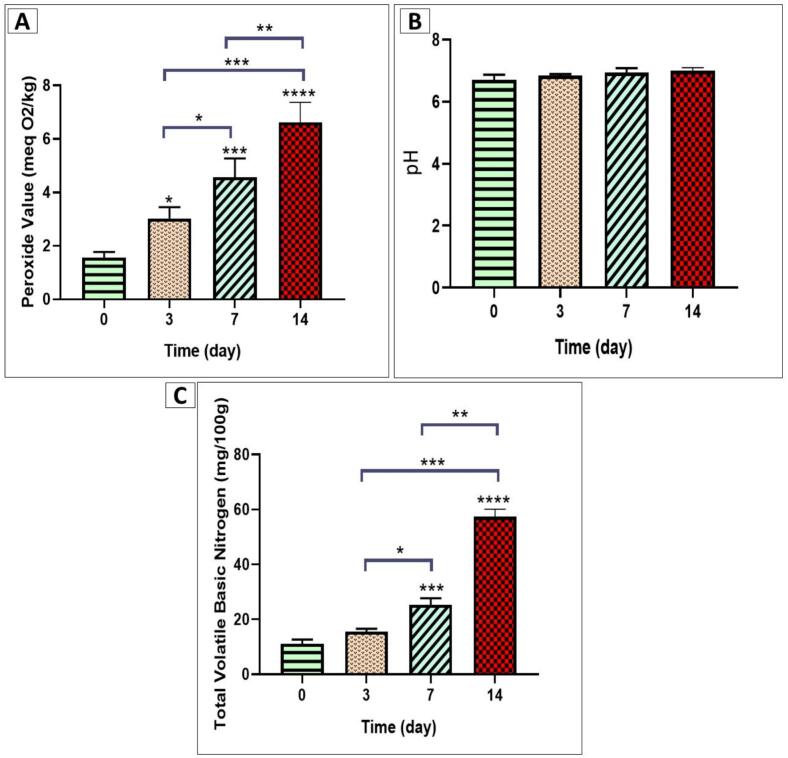
Chemical changes in fish samples during storage over 14 days. The peroxide value increased significantly over time, with a notable rise on day 14 compared to days 3 and 7 (A). pH values remained stable throughout the storage period, with no significant differences observed between days 0, 3, 7, and 14, indicating that pH may not be a sensitive indicator of fish quality deterioration under the given conditions (B). Total volatile basic nitrogen levels showed a progressive increase, especially on days 7 and 14, indicating protein degradation. A significant rise in total volatile basic nitrogen was also observed on day 14 compared to days 3 and 7, reflecting ongoing spoilage processes (C).

The results of chemical changes in the fish samples on days 0, 3, 7, and 14 are presented in Fig. 5.

#### 3.2.1 PV

The changes in PV showed an increasing trend on days 3, 7, and 14 compared to day 0, with statistical significance levels of (*p* < 0.05, *p* < 0.001, and *p* < 0.0001), respectively. These changes were accompanied by a significant increase in PV on day 14 compared to days 3 and 7, with significance levels of p < 0.001 and *p* < 0.01, respectively (Fig. 5A) (more information in Table 2).

#### 3.2.2 pH

No statistically significant differences were observed in the pH values of the fish samples throughout the storage period (days 0, 3, 7, and 14). This indicates that pH remained relatively stable over time, suggesting that it may not be a sensitive indicator for assessing the quality deterioration of the fish under the given storage conditions (Fig. 5B) (more information in Table 2).

#### 3.2.3 TVB-N

As shown in Fig. 5C (more information in Table 2), the TVB-N values exhibited a progressive increase, particularly noticeable on days 7 and 14 in comparison to day 0. These increases were statistically significant, with *p*-values of p < 0.05, p < 0.001, and p < 0.0001, respectively, indicating a clear trend of protein degradation over time. In parallel, the TVB-N also demonstrated a significant rise on day 14 when compared to days 3 and 7, with statistical significance levels of p < 0.001 and p < 0.01, respectively. These findings reflect ongoing oxidative and microbial spoilage processes during the storage period.

**Fig. 6:**
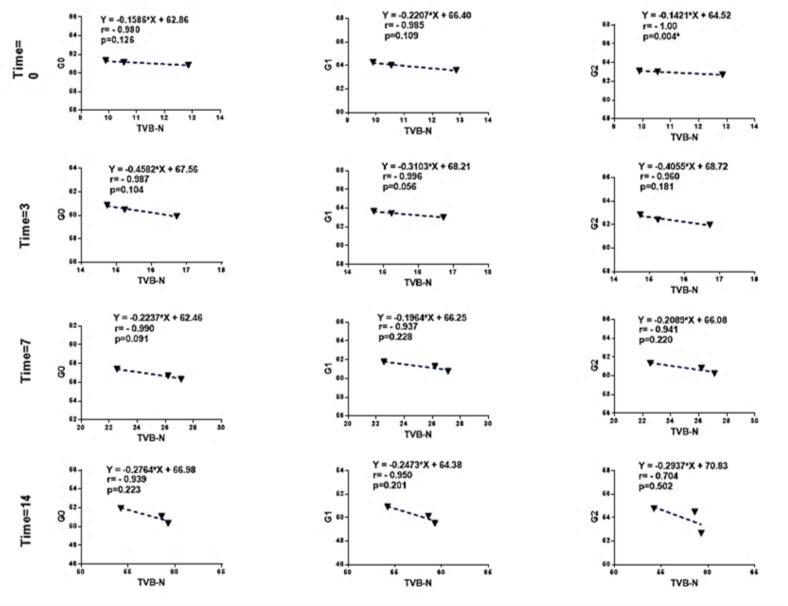
The scatter plots between the TVB-N and ΔE parameters at days 0, 3, 7, and 14 for all three groups (G0, G1, and G2) are shown. As observed, a strong inverse relationship between the TVB-N and ΔE parameters was reported, with the strongest and weakest correlations observed on the storage days for fish packaged at 4 °C. The correlation coefficients (r) are as follows: for day 0, G2 (*r* = −1.00) and G0 (*r* = −0.980); for day 3, G1 (*r* = −0.996) and G2 (*r* = −0.960); for day 7, G0 (*r* = −0.990) and G1 (*r* = −0.937); and for day 14, G1 (*r* = −0.950) and G2 (*r* = −0.704). It is worth noting that statistically, only a significant inverse relationship (*p* < 0.05) between the TVB-N and ΔE parameters was observed on day 0 for patch G2. This could be attributed to various factors, including the similar performance of the patches, the type and optimization of the patch formulation, the small sample size, and the optimization of the patch color, which requires further investigation in future studies.

To facilitate the visual comparison between the color parameter ΔE and the TVB-N parameter, scatter plots showing the relationship between TVB-N and ΔE at 0, 3, 7, and 14 days were plotted. As observed, the highest ΔE value was recorded on day 0, which showed a direct correlation with the low TVB-N values. As the storage time at 4 °C (refrigeration) progressed, TVB-N values increased, leading to a decrease in ΔE values in all three groups (G0, G1, and G2). This indicates an inverse relationship between the TVB-N and ΔE parameters in all three groups (Fig. 6).

The relationships between patch color change (ΔE) and spoilage indices (TVB-N, PV, and Microbial count) were evaluated using Pearson's correlation test, and the results are presented in the form of a table. As observed, a strong inverse relationship between the ΔE parameter and the TVB-N, PV, and Microbial count parameters was reported. The strongest inverse correlation between ΔE and TVB-N was found in group G1 (r = −0.996) on day 3, group G0 (r = −0.990) on day 7, and group G1 (r = −0.950) on day 14. The strongest inverse correlation between ΔE and the number of Psychrophiles on days 3, 7, and 14 was observed in group G2 (r = −1.00), G1 (*r* = −0.947), and G1 (*r* = −0.986), respectively. The strongest inverse correlation between ΔE and the number of Thermophilic bacteria on days 3, 7, and 14 was found in group G1, while the strongest inverse correlation between ΔE and PV on days 3, 7, and 14 was also reported in group G1 (Table 1).

**Table 1:** Results of the correlation analysis between the spoilage indicators and the color change of the patch.

Parameters	Time (day)	ΔE		
		G0	G1	G2
TVB-N	0	*r* = − 0.980 *p* = 0.126	*r* = − 0.985 *p* = 0.109	*r* = − 1.00 *p* = 0.004^⁎^
	3	*r* = − 0.987 *p* = 0.104	*r* = − 0.996 *p* = 0.056	*r* = − 0.960 *p* = 0.181
	7	*r* = − 0.990 *p* = 0.091	*r* = − 0.937 *p* = 0.228	*r* = − 0.941 *p* = 0.220
	14	*r* = − 0.939 *p* = 0.223	*r* = − 0.950 *p* = 0.201	*r* = − 0.704 *p* = 0.502

Psychrophiles	0	r = − p = −	r = − p = −	r = − p = −
	3	*r* = − 0.989 *p* = 0.097	*r* = − 0.974 *p* = 0.144	r = −1.00 *p* = 0.019^⁎^
	7	*r* = − 0.856 *p* = 0.346	*r* = − 0.947 *p* = 0.209	*r* = −0.943 *p* = 0.217
	14	*r* = − 0.979 *p* = 0.129	r = − 0.986 *p* = 0.107	*r* = −0.801 *p* = 0.408

Thermophilic	0	r = − 0.947 *p* = 0.208	*r* = − 0.938 *p* = 0.224	*r* = −0.863 *p* = 0.337
	3	*r* = − 0.973 *p* = 0.147	*r* = − 0.988 *p* = 0.099	r = −0.938 *p* = 0.225
	7	*r* = − 0.970 *p* = 0.157	r = − 0.999 *p* = 0.020^⁎^	r = −0.999 *p* = 0.028^⁎^
	14	*r* = − 0.928 *p* = 0.243	*r* = − 0.940 *p* = 0.221	*r* = −0.682 *p* = 0.522

PV	0	r = − 0.986 *p* = 0.106	r = − 0.990 *p* = 0.089	*r* = −0.999 *p* = 0.024^⁎^
	3	*r* = − 0.986 *p* = 0.106	r = − 0.996 *p* = 0.058	*r* = −0.959 *p* = 0.183
	7	*r* = − 0.964 *p* = 0.171	r = − 0.999 *p* = 034^⁎^	*r* = −0.998 *p* = 042^⁎^
	14	*r* = − 0.963 *p* = 0.173	*r* = − 0.972 *p* = 0.151	*r* = −0.758 *p* = 0.452

*Significant at the level of 0.05. r = correlation coefficient value. The relationship between patch color change and spoilage indices was assessed using Pearson's correlation test. As observed, our findings indicated a strong inverse relationship between color changes (ΔE) and spoilage indices (TVB-N, PV, and Microbial count) for the G1 patch in most cases, demonstrating the high sensitivity and effectiveness of this patch in monitoring spoilage ofpackaged fish stored at refrigeration temperature (4 °C). Additionally, according to the table above, on day 3, a statistically significant inverse relationship was observed only between the number of Psychrophiles and ΔE in the G2 patch (*p* < 0.05). On day 7, we also found a statistically significant inverse relationship between the number of Thermophilic bacteria and ΔE in the G2 patch, as well as a significant inverse relationship between the PV spoilage parameter and ΔE in both G1 and G2 patches (p < 0.05). Based on the data presented in the table, it is evident that a strong inverse relationship exists between ΔE and the spoilage parameters, although the significance level was relatively low. This may be attributed to various factors, including the similar performance of the patches, the type and optimization of the patch formulation, the small sample size, and the optimization of patch color, which warrants further investigation in future studies.

### 3.3 Microbiological analyses

**Fig. 7:**
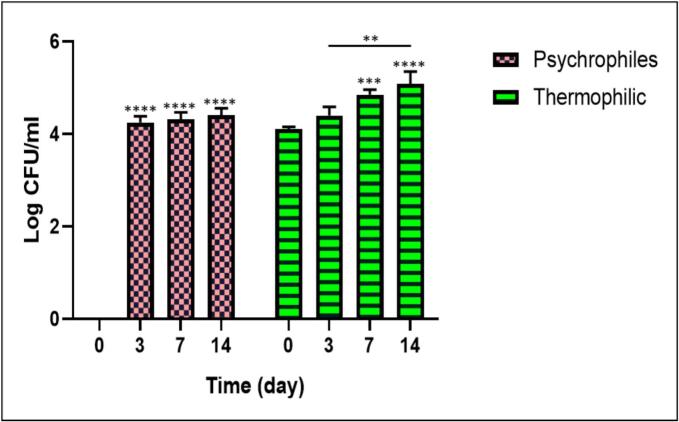
Microbiological analysis of Beluga (*Huso huso*) fish samples was conducted to assess changes in the populations of psychrophilic and thermophilic bacteria on days 0, 3, 7, and 14. Values are reported as Mean ± SD; * Significant at the level of 0.05. (** p < 0.01), (*** p < 0.001), and (**** p < 0.0001).

The changes in the populations of *Psychrophilic* and *Thermophilic* bacteria on days 0, 3, 7, and 14 in three replicates are reported in Fig. 7 (more information in Table 2). On day 0, no growth of *Psychrophilic* bacteria was observed, and the initial *Psychrophilic* bacterial count in the packaged fillets was 0, while the count for *Thermophilic* bacteria was, on average, Log CFU/ml 4.1 (Replicate 1: 4.041, Replicate 2: 4.146, and Replicate 3: 4.113). On day 3, as expected, the microbial load increased, with the average *Psychrophilic* bacterial count being Log CFU/ml 4.249 (Replicate 1: 4.113, Replicate 2: 4.255, and Replicate 3: 4.38), and the average *Thermophilic* bacterial count being Log CFU/ml 4.396 (Replicate 1: 4.255, Replicate 2: 4.322, and Replicate 3: 4.612). On day 7, the average microbial load for both *Psychrophilic* and *Thermophilic* bacteria was Log CFU/ml 4.316 and 4.845, respectively, and on day 14, it was Log CFU/ml 4.402 and 5.079.

**Table 2:** Summary of mean values and standard deviations of PV, TVB-N, pH, microbial counts, and sensory scores at different time points.

Peroxide Value (PV)							
Comparison	Mean 1	Mean 2	Mean Diff.	SE of Diff.	SD (approx.)	Adjusted *P* Value	Significance
Day 0 VS. Day 3	1.567	3.033	−1.467	0.457	0.792	0.0495	*
Day 0 VS. Day 7	1.567	4.567	−3	0.457	0.792	0.0008	***
Day 0 VS. Day 14	1.567	6.633	−5.067	0.457	0.792	<0.0001	****
Day 3 VS. Day 7	3.033	4.567	−1.533	0.457	0.792	0.0404	*
Day 3 VS. Day 14	3.033	6.633	−3.6	0.457	0.792	0.0002	***
Day 7 VS. Day 14	4.567	6.633	−2.067	0.457	0.792	0.0084	**

**Total Volatile Basic Nitrogen (TVB-N)**							
Day 0 VS. Day 3	11.09	15.57	−4.473	1.673	2.893	0.1053	ns
Day 0 VS. Day 7	11.09	25.29	−14.2	1.673	2.893	0.0001	***
Day 0 VS. Day 14	11.09	57.37	−46.27	1.673	2.893	<0.0001	****
Day 3 VS. Day 7	15.57	25.29	−9.723	1.673	2.893	0.0018	**
Day 3 VS. Day 14	15.57	57.37	−41.8	1.673	2.893	<0.0001	****
Day 7 VS. Day 14	25.29	57.37	−32.08	1.673	2.893	<0.0001	****

**pH**							
Day 0 VS. Day 3	6.7	6.833	−0.1333	0.1054	0.182	0.6073	ns
Day 0 VS. Day 7	6.7	6.933	−0.2333	0.1054	0.182	0.1991	ns
Day 0 VS. Day 14	6.7	7	−0.3	0.1054	0.182	0.0827	ns
Day 3 VS. Day 7	6.833	6.933	−0.1	0.1054	0.182	0.7807	ns
Day 3 VS. Day 14	6.833	7	−0.1667	0.1054	0.182	0.4392	ns
Day 7 VS. Day 14	6.933	7	−0.06667	0.1054	0.182	0.9187	ns

**Sensory Scores (Color)**							
Day 0 VS. Day 3	5	5	0	0.1947	0.337	>0.9999	ns
Day 0 VS. Day 7	5	4.667	0.3333	0.1947	0.337	0.3344	ns
Day 0 VS. Day 14	5	3.433	1.567	0.1947	0.337	<0.0001	****
Day 3 VS. Day 7	5	4.667	0.3333	0.1947	0.337	0.3344	ns
Day 3 VS. Day 14	5	3.433	1.567	0.1947	0.337	<0.0001	****
Day 7 VS. Day 14	4.667	3.433	1.233	0.1947	0.337	<0.0001	****

**Sensory Scores (Smell)**							
Day 0 VS. Day 3	5	4.567	0.4333	0.1947	0.337	0.138	ns
Day 0 VS. Day 7	5	4.033	0.9667	0.1947	0.337	0.0001	***
Day 0 VS. Day 14	5	0	5	0.1947	0.337	<0.0001	****
Day 3 VS. Day 7	4.567	4.033	0.5333	0.1947	0.337	0.0468	*
Day 3 VS. Day 14	4.567	0	4.567	0.1947	0.337	<0.0001	****
Day 7 VS. Day 14	4.033	0	4.033	0.1947	0.337	<0.0001	****

**Sensory Scores (Texture)**							
Day 0 VS. Day 3	5	5	0	0.1947	0.337	>0.9999	ns
Day 0 VS. Day 7	5	4.767	0.2333	0.1947	0.337	0.6324	ns
Day 0 VS. Day 14	5	3.333	1.667	0.1947	0.337	<0.0001	****
Day 3 VS. Day 7	5	4.767	0.2333	0.1947	0.337	0.6324	ns
Day 3 VS. Day 14	5	3.333	1.667	0.1947	0.337	<0.0001	****
Day 7 VS. Day 14	4.767	3.333	1.433	0.1947	0.337	<0.0001	****

**Sensory Scores (Acceptability)**							
Day 0 VS. Day 3	5	4.833	0.1667	0.1947	0.337	0.8272	ns
Day 0 VS. Day 7	5	4.433	0.5667	0.1947	0.337	0.0315	*
Day 0 VS. Day 14	5	2.933	2.067	0.1947	0.337	<0.0001	****
Day 3 VS. Day 7	4.833	4.433	0.4	0.1947	0.337	0.19	ns
Day 3 VS. Day 14	4.833	2.933	1.9	0.1947	0.337	<0.0001	****
Day 7 VS. Day 14	4.433	2.933	1.5	0.1947	0.337	<0.0001	****

**Microbial Counts (Psychrophilic (PS) and Thermophilic Bacteria (TH))**							
Day 0 (PS) VS. Day 0 (TH)	0	4.1	−4.1	0.1255	0.217	<0.0001	****
Day 0 (PS) VS. Day 3 (PS)	0	4.249	−4.249	0.1255	0.217	<0.0001	****
Day 0 (PS) VS. Day 3 (TH)	0	4.396	−4.396	0.1255	0.217	<0.0001	****
Day 0 (PS) VS. Day 7 (PS)	0	4.317	−4.317	0.1255	0.217	<0.0001	****
Day 0 (PS) VS. Day 7 (TH)	0	4.846	−4.846	0.1255	0.217	<0.0001	****
Day 0 (PS) VS. Day 14 (PS)	0	4.403	−4.403	0.1255	0.217	<0.0001	****
Day 0 (PS) VS. Day 14 (TH)	0	5.08	−5.08	0.1255	0.217	<0.0001	****
Day 0 (TH) VS. Day 3 (PS)	4.1	4.249	−0.1493	0.1255	0.217	0.9997	ns
Day 0 (TH) VS. Day 3 (TH)	4.1	4.396	−0.2963	0.1255	0.217	0.5883	ns
Day 0 (TH) VS. Day 7 (PS)	4.1	4.317	−0.2167	0.1255	0.217	0.953	ns
Day 0 (TH) VS. Day 7 (TH)	4.1	4.846	−0.7457	0.1255	0.217	0.0006	***
Day 0 (TH) VS. Day 14 (PS)	4.1	4.403	−0.3027	0.1255	0.217	0.5514	ns
Day 0 (TH) VS. Day 14 (TH)	4.1	5.08	−0.9797	0.1255	0.217	<0.0001	****
Day 3 (PS) VS. Day 3 (TH)	4.249	4.396	−0.147	0.1255	0.217	0.9998	ns
Day 3 (PS) VS. Day 7 (PS)	4.249	4.317	−0.06733	0.1255	0.217	>0.9999	ns
Day 3 (PS) VS. Day 7 (TH)	4.249	4.846	−0.5963	0.1255	0.217	0.006	**
Day 3 (PS) VS. Day 14 (PS)	4.249	4.403	−0.1533	0.1255	0.217	0.9995	ns
Day 3 (PS) VS. Day 14 (TH)	4.249	5.08	−0.8303	0.1255	0.217	0.0002	***
Day 3 (TH) VS. Day 7 (PS)	4.396	4.317	0.07967	0.1255	0.217	>0.9999	ns
Day 3 (TH) VS. Day 7 (TH)	4.396	4.846	−0.4493	0.1255	0.217	0.0676	ns
Day 3 (TH) VS. Day 14 (PS)	4.396	4.403	−0.006333	0.1255	0.217	>0.9999	ns
Day 3 (TH) VS. Day 14 (TH)	4.396	5.08	−0.6833	0.1255	0.217	0.0015	**
Day 7 (PS) VS. Day 7 (TH)	4.317	4.846	−0.529	0.1255	0.217	0.0182	*
Day 7 (PS) VS. Day 14 (PS)	4.317	4.403	−0.086	0.1255	0.217	>0.9999	ns
Day 7 (PS) VS. Day 14 (TH)	4.317	5.08	−0.763	0.1255	0.217	0.0004	***
Day 7 (TH) VS. Day 14 (PS)	4.846	4.403	0.443	0.1255	0.217	0.0749	ns
Day 7 (TH) VS. Day 14 (TH)	4.846	5.08	−0.234	0.1255	0.217	0.905	ns
Day 14 (PS) VS. Day 14 (TH)	4.403	5.08	−0.677	0.1255	0.217	0.0017	**

The increase in microbial load could be attributed to the growth of bacteria such as *Pseudomonas, Escherichia coli, Acinetobacter, and Shewanella* during the storage period. The population changes of *Psychrophiles* on days 3, 7, and 14 showed a significant increase compared to day 0 (*p* < 0.001). The increase in *Thermophilic* populations on days 7 and 14 compared to day 0 was also significant (p < 0.001 and *p* < 0.0001), respectively. A further significant increase (*p* < 0.01) was observed on day 14 compared to day 7 in *Psychrophiles* populations.

### 3.4 Sensory analyses

**Fig. 8:**
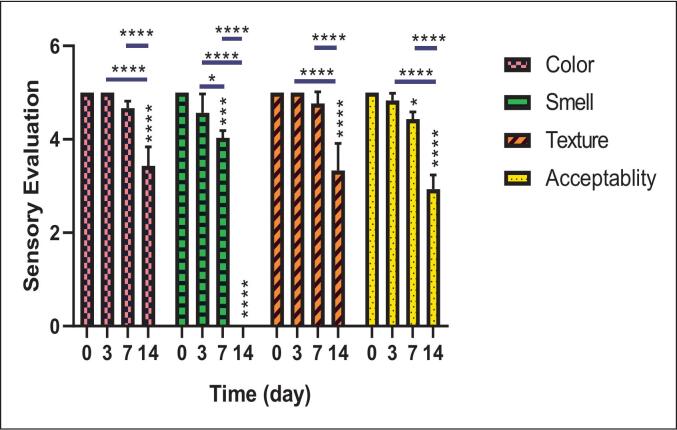
Sensory analyses of Beluga (*Huso huso*) fish samples for color, smell, texture, and acceptability. Values are reported as Mean ± SD; * Significant at the level of 0.05. (* *p* < 0.05), (*** p < 0.001), and (**** p < 0.0001).

According to the sensory analysis results of the studied samples presented in Fig. 8 (more information in Table 2), the color of the fish showed a significant decrease on day 14 compared to days 0, 3, and 7 (p < 0.0001). Similarly, changes related to smell exhibited a significant reduction on days 7 and 14 in comparison with day 0, recorded as (p < 0.001 and p < 0.0001), respectively. This decrease was also statistically significant when comparing day 14 with days 3 and 7 (p < 0.0001). Regarding texture, a significant decrease was observed on day 14 compared to days 3 and 7 (p < 0.0001). The changes in acceptability also indicated a significant decline on day 14 compared to days 0, 3, and 7 (p < 0.0001).

## 4 Discussion

In this study, a unique approach using pH-sensitive indicator patches based on polymer nanofibers and anthocyanins to monitor the freshness of Beluga Fish (*Huso huso*) fillets was evaluated. These patches visually respond to pH changes that occur during spoilage, providing a reliable, real-time indication of freshness. The combination of anthocyanins extracted from red cabbage, a natural colorant, with nanofiber technology, offers an innovative, eco-friendly solution for monitoring food quality.

Unlike traditional methods, this approach allows direct observation of spoilage progression, making it a novel tool for food safety and quality control. The effectiveness of pH-sensitive indicator patches for monitoring the freshness of Beluga Fish (*Huso huso*) fillets during storage at 4 °C was also evaluated through a combination of visual observations, instrumental measurements, and analytical techniques. The results clearly showed that the pH-responsive nanofiber patches, in combination with the color derived from anthocyanins, visibly responded to pH changes associated with spoilage. This response made them a valuable tool for tracking the freshness of Beluga sturgeon fish fillets.

The three experimental groups, G0, G1, and G2, exhibited distinct performance characteristics under varying pH conditions (pH 5–9). By day three of storage at 4 °C, all patches showed observable color changes, with groups G0 and G1 exhibiting greater visual clarity than group G2. The superior performance of G0 and G1 can be attributed to differences in formulation, specifically their ability to absorb amine compounds released during spoilage, which raise pH levels. Notably, group G1 exhibited sharper, more defined color changes than G0, indicating greater sensitivity to spoilage induced alterations. This finding aligns with the observation that patches made with a combination of polymer nanofibers and anthocyanins responded more reliably to changes in the spoilage process.

On the contrary, the G2 patches, made with PCL, showed reduced clarity, particularly due to PCL's lower moisture sensitivity. This prevented effective absorption of volatile amines, which likely limited the color response of these patches to changes in pH. Additionally, the lack of protective measures during the incorporation of anthocyanins into G2 patches may have contributed to their diminished performance. These findings underscore the critical role of formulation and material choice in the efficiency of pH-sensitive indicators. As evidenced by the visual results, patches in groups G0 and G1 maintained higher clarity throughout the storage period, with G1 being more responsive than G0, reflecting its formulation's enhanced sensitivity to pH shifts.

Instrumental color analysis further confirmed the visual observations. Both the a* and L* values showed a clear pattern of change with increasing pH, indicating the absorption of amine compounds and shifts in the spoilage process. Specifically, the a* values, which indicate redness, decreased as pH increased, showing a clear trend of pH-induced alterations. The reduction in L* values across all groups was consistent with the transformation of chalcone precursors into darker brown pigments, a process that corresponds with the degradation of yellow chalcones. The color changes were evident on day 14, where G0 exhibited a dark yellowish-brown hue, G1 showed a dull yellow-brown, and G2 appeared light yellow-brown, indicating different stages of spoilage. These results align with the observed increases in TVB-N, a key indicator of protein degradation during spoilage.

The relationship between color changes and TVB-N levels was particularly noteworthy. As TVB-N levels progressively increased throughout the storage period, so too did the color transitions in the patches, confirming that the pH-sensitive labels provided an accurate and reliable indication of fish quality deterioration. TVB-N, which reflects microbial and enzymatic degradation of fish proteins, has long been used as an indicator of spoilage, with elevated levels signifying bacterial activity and degradation. In this study, TVB-N levels increased significantly on days 7 and 14, mirroring the visible color changes observed in the indicator patches. The rise in TVB-N values, especially between days 7 and 14, confirmed the onset of significant spoilage and microbial growth, which the pH indicator patches were able to track effectively.

Microbiological analyses further supported the correlation between color changes and spoilage. Psychrophilic and thermophilic bacterial populations showed significant growth over the course of the storage period, with the highest levels recorded on days 7 and 14. These microbial increases mirrored the changes in TVB-N levels and were consistent with the visible color shifts in the pH indicator patches. Bacterial species such as *Pseudomonas, Escherichia coli*, *Acinetobacter*, and *Shewanella* are known to play a key role in the spoilage process of fish. The correlation between microbial load, TVB-N, and color changes in the indicator labels supports the hypothesis that pH-sensitive patches are an effective tool for real-time monitoring of fish freshness and spoilage.

Sensory analyses also reflected the deterioration of fish quality, with significant declines in color, odor, texture, and overall acceptability observed, particularly on day 14. The reduction in sensory scores further corroborated the instrumental and microbiological findings, confirming that the fish had surpassed the threshold of acceptable freshness by the end of the storage period. This emphasizes the utility of pH-sensitive indicator patches not only as an analytical tool but also as a means to complement sensory evaluation in assessing food quality.

For instance, a study focused on the development of a pH-sensitive label for real-time monitoring of chicken meat freshness (Riahi et al., 2019). The results showed that Bromocresol Green (BCG), a pH-sensitive color indicator, effectively detected chicken spoilage by being attached to the inner side of the packaging. Chemical (pH and TVB-N), microbiological, and sensory analyses were conducted at intervals of 0, 3, 5, 7, 8, and 10 days of storage at 4 °C. On day one, TVB-N was 19 mg per 100 g, which increased to 29 mg per 100 g by day eight and reached 46 mg per 100 g by day ten. The results revealed that color changes in the BCG indicator, associated with chicken spoilage, were visually detectable. On day ten, the indicator color changed to blue, and the total bacterial count reached approximately 7.20 Log10 CFU/g. A strong correlation was found between TVB-N, pH, microbiological and sensory analyses, and the color changes of the indicator during storage, demonstrating its reliability for spoilage tracking (Riahi et al., 2019).

A freshness indicator for fish was developed to monitor quality during storage by detecting ammonia levels among the TVB-N compounds (Kim et al., 2023). The indicator changed color in response to pH shifts caused by ammonia release, reflecting the spoilage process. Cherry red and bromocresol purple were selected as the indicator colorants, and eco-DEHCH and Breathron films were incorporated to enhance environmental and economic viability. During storage experiments, fish were kept at varying temperatures, and parameters such as pH, TVB-N, ammonia content, and bacterial count were monitored. The results showed significant color changes, from yellow to black, as spoilage progressed, with the color turning purple at advanced stages of decay. These changes were in direct correlation with increases in TVB-N, pH, and bacterial counts, confirming the indicator's reliability for tracking spoilage (Kim et al., 2023).

In the case of a pH-sensitive sensor based on bacterial cellulose nanofibers and black carrot anthocyanins (CA), the indicator demonstrated clear and noticeable color changes from red to gray across a pH range of 2 to 11 (Moradi et al., 2019). The indicator was able to differentiate between various stages of fish freshness, with dark carmine indicating freshness, attractive pink for optimal consumption, and blue or earthy jelly bean colors signaling spoilage. Strong positive correlations were observed between color differences and both bacterial counts (*R* = 0.952 and 0.991) and TVB-N (*R* = 0.815 and 0.92) for rainbow trout and common carp. These results highlighted the potential of the bacterial cellulose-based pH sensor for real-time spoilage monitoring of fish, confirming its effectiveness as a tool for assessing fish freshness (Moradi et al., 2019).

Lastly, the use of pH-sensitive color indicators in smart packaging systems was tested for monitoring the spoilage of Pangasius fillets at room temperature (Ningtyas et al., 2023). The goal was to assess the effectiveness of methyl orange (MO), methyl red (MR), and bromocresol green (BCG) as indicators for detecting quality degradation in Pangasius fillets. Sensitivity tests using ammonia gas (NH₄OH) showed that all three indicators responded to pH changes. Field experiments were conducted over 15 h, with the color changes of the indicators being monitored every three hours. The freshness threshold for Pangasius was identified between the 6th and 9th hours of storage, with significant color changes occurring at the 9-h mark. MO shifted from pink to yellowish-orange, MR from deep red to yellowish-orange, and BCG from light yellow to blue. These color changes were corroborated by pH, total plate count, and sensory evaluations, confirming the suitability of these pH-sensitive indicators for smart packaging systems. Among them, the BCG indicator demonstrated superior sensitivity to freshness changes, outperforming the other two indicators (Ningtyas et al., 2023).

The study also investigated the development of a smart pH-monitoring label based on bacterial cellulose (BC) nanofibers infused with anthocyanins from *Brassica oleracea* (red cabbage) (Pourjavaher et al., 2017). This label was tested for its response to pH changes over a range of pH from 2 to 10, with clear color transitions observed across this range. The label's performance was influenced by the concentration of anthocyanins, with a noticeable reduction in the mechanical strength of the label as the anthocyanin concentration increased. However, labels with diluted anthocyanins showed better color responses and were more flexible and moisture-absorbent, indicating their potential for use in food packaging. FT-IR analysis indicated chemical interactions between the BC nanofibers and anthocyanins, while SEM observations revealed the degradation of cellulose microfibrils at higher anthocyanin concentrations. The results demonstrated that the pH-sensitive labels had strong potential as an eco-friendly and low-cost smart packaging solution for monitoring food freshness, particularly for perishable products. The color changes provided a clear visual cue for pH fluctuations, making these labels a promising tool for tracking spoilage during food storage (Pourjavaher et al., 2017).

In the following section, we compare the G0, G1, and G2 patches with several existing pH indicators. Table 3 provides a comprehensive comparison of various features, including indicator type, fabrication method, response time, pH range, and color change (ΔE). The results show that the G0 and G1 patches exhibit faster response times and more distinct color changes than other pH indicators. Specifically, the G1 patches using the PCL/PVA/GG combination demonstrated higher sensitivity to pH changes and a lower ΔE, indicating better performance than the other groups.

**Table 3:** Comparison of pH indicator patches (G0, G1, G2) with existing pH indicators based on key performance parameters.

Title	Application	Indicator Type	Fabrication Method	Matrix	Response time	pH range	ΔE
**Our Current Study**	Monitoring the Quality of Beluga Fish (*Huso huso*)	Red cabbage anthocyanins (RCAs)	Co-Spining	PCL/PVA/CMC	Fast (Instant color response)	5–9	<61
			Co-Spining	PCL/PVA/GG	Fast (instant color response)		<64
			Electrospinning/ Immersion	PCL	Slower response than the previous two groups		<63
Development of a colorimetric pH indicator using nanofibers containing *Spirulina* sp. LEB 18 (Kuntzler et al., 2020)	Intelligent packaging	Microalgae biomass *Spirulina* sp. LEB 18	Electrospinning	Polylactic acid and polyethylene oxide	Fast (instant color response due to hydrophilicity)	1–10	<12
Preparation and characterization of intelligent packaging labels based on pea starch, κ-carrageenan and black raspberry extract for monitoring freshness of pork (Jiang et al., 2024)	Monitoring freshness of pork	Black raspberry extract	Incorporation into pea starch/κ-carrageenan matrix (casting method)	Pea starch, κ-carrageenan	Fast	2–13	<11
Preparation of an intelligent film based on chitosan/oxidized chitin nanocrystals incorporating black rice bran anthocyanins for seafood spoilage monitoring (Wu et al., 2019)	Fresh fish and shrimp	Black rice bran anthocyanins	Immobilization of BACNs in chitosan/oxidized chitin nanocrystals matrix (casting method)	Chitosan/oxidized chitin nanocrystals	High sensitivity to pH and antioxidant properties	2–12	<23
Halochromic (pH-Responsive) Indicators Based on Natural Anthocyanins for Monitoring Fish Freshness/Spoilage (Khezerlou et al., 2023)	Fish fillets	Saffron petal anthocyanins (SPA) and barberry anthocyanins (BA)	Encapsulation in gelatin/chitin nanofiber films (casting method)	Gelatin/chitin	Real-time (visible changes prior to consumption)	2–12	<53
A colorimetric film based on polyvinyl alcohol/sodium carboxymethyl cellulose incorporated with red cabbage anthocyanin for monitoring pork freshness (Liu et al., 2021)	Pork	Red cabbage anthocyanins (RCAs)	Solvent evaporation of PVOH/CMC-Na with RCAs	Polyvinyl alcohol/sodium carboxymethyl cellulose	High (RCAs provide high sensitivity to ammonia)	2–12	<36

While this study provides valuable insights into the use of pH-sensitive indicator patches to track the freshness of fish fillets, it is important to acknowledge several limitations. One significant limitation is the failure to evaluate the actual shelf life of the fish fillets during extended storage. The impact of real-life storage conditions and varying temperatures on the patches' performance was not assessed. Additionally, sensory evaluation was conducted only at selected time points, which may not have captured the full spectrum of sensory changes during spoilage.

Although the pH-sensitive electrospun patches showed good sensitivity under laboratory conditions, their durability was not evaluated. This means that the long-term stability of the color response during storage and in real distribution conditions remains unknown. Secondly, the experiments were conducted in controlled laboratory environments and using a single food matrix (Beluga sturgeon), which limits generalizability to other seafood or meat products.

Despite these limitations, the combination of visual clarity, pH sensitivity, and correlation with microbial and chemical indicators makes pH-sensitive labels an attractive choice for innovative food packaging applications. In this study, the pH indicator patches successfully tracked the spoilage of fish fillets, providing a simple and effective means to monitor the quality of seafood products. The results underscore the potential of pH-sensitive indicator patches as a practical and reliable tool for monitoring the freshness and spoilage of fish. These indicators offer a promising solution for the seafood industry, providing real-time, non-invasive monitoring of food quality.

Future research could focus on optimizing the formulation to enhance the sensitivity and responsiveness of the patches. It would also be valuable to extend their application to other perishable food products, such as poultry and dairy. Furthermore, addressing the study's limitations by evaluating the long-term shelf life of fish and testing the patches under various environmental conditions would help better understand their applicability in commercial settings. The development of such innovative packaging systems holds great promise for improving food safety, reducing waste, and enhancing consumer confidence in the quality of perishable food products.

## 5 Conclusion

Since the polymers used in the formulation of the patches determine the nanofiber structure and affect key properties such as moisture absorption, mechanical stability, pH sensitivity (due to fish spoilage and the production of volatile amines assessed in the TVB-N test), and color release, the polymer matrices in the designed nanofiber patches play a critical role in the performance of the pH indicator. For example, PCL, used in the structure of all three patches, forms the primary scaffold and provides adequate mechanical stability. However, due to its hydrophobic nature, PCL exhibits low moisture absorption, which impairs the uniform loading of the extract, the absorption of volatile compounds from the fish surface, and the release of the color indicator, thus reducing the pH sensor's sensitivity.

To address this, we suggest modifying the PCL polymer matrix by introducing functional groups to increase surface roughness. This modification would enhance contact with ammonia vapors (produced by fish spoilage) and improve the pH indicator's sensitivity, although further investigation is needed. Additionally, combining PCL with other hydrophilic polymers, such as PVA, CMC, and GG, has helped to mitigate some of these issues by increasing moisture absorption and enhancing pH sensitivity. This combination improves extract absorption and pH responsiveness. However, the combination of CMC and PVA may lead to excessive swelling and patch degradation in humid environments, necessitating further studies and optimization of the polymer matrix to ensure its stability under high-moisture conditions.

The inclusion of GG in the polymer matrix improves the gel-like structure and uniformity of the nanofibers and increases patch swelling. However, it needs to be combined with other polymers, such as PVA, to improve electrospinning properties. PVA, a hydrophilic polymer with high spinnability, produces nanofibers with a high surface-to-volume ratio, facilitating the encapsulation of pH indicators and increasing sensitivity to environmental changes. However, excessive swelling in moisture and the need for cross-linking agents to ensure long-term stability may affect the patch's performance.

Thus, as observed, the combination of these polymers can create a synergistic effect, but optimizing their ratios within the polymer matrix and ensuring their stability against moisture and heat is essential to achieve a balance between the advantages and limitations.

In this study, pH-sensitive indicator patches were successfully developed and tested for monitoring the freshness of Beluga Fish (*Huso huso*) fillets during storage at 4 °C. The results demonstrated that the patches exhibited clear, distinguishable color changes corresponding to increased pH, indicative of spoilage caused by microbial and chemical degradation. Among the three experimental groups (G0, G1, and G2), the patches from group G1 exhibited the most pronounced and reliable color transitions, making them the most suitable for innovative packaging applications for fish products.

Instrumental analysis of color values, along with sensory and microbiological data, confirmed the relationship between the observed color changes and the degradation processes of the fish, such as protein breakdown and bacterial growth. The patches' ability to visually reflect the freshness and spoilage stages aligns well with changes in TVB-N levels, microbial counts, and sensory properties, providing a simple and effective means of tracking food quality. These findings highlight the potential of pH-sensitive indicator patches as a low-cost, non-invasive tool for monitoring fish quality during storage, offering a promising approach to improve food safety and reduce waste.

The key feature of this patch is its direct attachment to the fish's surface, which significantly enhances its sensitivity. Unlike traditional patches that are placed beneath food packaging films and do not make direct contact, this design allows for more precise monitoring. Furthermore, due to direct contact, all materials used in the formulation are both safe and natural. A portion of the formulation is already used in the food industry as an ingredient. At the same time, the remaining materials are biocompatible, making the patch not only effective but also in line with food safety and health standards.

Future research should focus on optimizing the formulation of pH-sensitive indicator patches to improve their sensitivity and responsiveness to spoilage-related pH shifts. This could involve exploring alternative nanofiber materials or colorants that offer enhanced performance. Additionally, further investigations are needed to assess the real-world applicability of these patches by testing them under varied storage conditions and across different food products, such as poultry and dairy, to confirm their versatility. Finally, a deeper exploration of the mechanisms underlying pH shifts during spoilage and their correlation with microbial growth could further refine the design and functionality of the patches, enabling more precise, real-time monitoring of food quality.

## Availability of data and materials

All data analyzed during this study are included in this published article and supporting information.

## CRediT authorship contribution statement

**Seyed Mohammad Ali Ebnetorab:** Writing – original draft, Investigation, Conceptualization. **Hamed Ahari:** Writing – review & editing, Visualization, Validation, Project administration, Methodology, Investigation, Data curation, Conceptualization. **Seid Mahdi Jafari:** Writing – review & editing, Visualization, Formal analysis, Data curation. **Maryam Mizani:** Writing – review & editing, Resources, Investigation, Conceptualization. **Seyed Amir Ali Anvar:** Writing – review & editing, Validation, Methodology, Investigation, Conceptualization.

## Funding

This research did not receive any specific grant from funding agencies in the public, commercial, or not-for-profit sectors.

## Declaration of competing interest

The authors declare that they have no known competing financial interests or personal relationships that could have appeared to influence the work reported in this paper.

## Data Availability

All data is available in article
